# Site-Specific Cleavage by Topoisomerase 2: A Mark of the Core Centromere

**DOI:** 10.3390/ijms19020534

**Published:** 2018-02-10

**Authors:** Walter E. Mills, Jennifer M. Spence, Tatsuo Fukagawa, Christine J. Farr

**Affiliations:** 1 Department of Genetics, University of Cambridge, Downing St, Cambridge CB2 3EH, UK; w_e_mills@yahoo.co.uk (W.E.M.); jennyspence44@yahoo.co.uk (J.M.S.); 2 Laboratory of Chromosome Biology, Graduate School of Frontier Biosciences, Osaka University, Suita, Osaka 565-0871, Japan; tfukagawa@fbs.osaka-u.ac.jp

**Keywords:** topoisomerase 2α (topo 2α), centromere, mitosis, etoposide, cleavage, secondary DNA structure

## Abstract

In addition to its roles in transcription and replication, topoisomerase 2 (topo 2) is crucial in shaping mitotic chromosomes and in ensuring the orderly separation of sister chromatids. As well as its recruitment throughout the length of the mitotic chromosome, topo 2 accumulates at the primary constriction. Here, following cohesin release, the enzymatic activity of topo 2 acts to remove residual sister catenations. Intriguingly, topo 2 does not bind and cleave all sites in the genome equally; one preferred site of cleavage is within the core centromere. Discrete topo 2-centromeric cleavage sites have been identified in α-satellite DNA arrays of active human centromeres and in the centromere regions of some protozoans. In this study, we show that topo 2 cleavage sites are also a feature of the centromere in *Schizosaccharomyces pombe*, the metazoan *Drosophila melanogaster* and in another vertebrate species, *Gallus gallus* (chicken). In vertebrates, we show that this site-specific cleavage is diminished by depletion of CENP-I, an essential constitutive centromere protein. The presence, within the core centromere of a wide range of eukaryotes, of precise sites hypersensitive to topo 2 cleavage suggests that these mark a fundamental and conserved aspect of this functional domain, such as a non-canonical secondary structure.

## 1. Introduction

Topoisomerase 2 (topo 2) is an essential enzyme that controls DNA topology and contributes to many aspects of DNA metabolism, including replication and transcription [1,2]. In mitosis, topo 2 is present throughout the entire length of the chromosome, where it plays a crucial role in shaping and maintaining chromosome structure and in segregation. In addition, in vertebrate cells, it is well established, from numerous studies on both fixed and live cells, that topo 2α (one of two isoforms present in vertebrates) accumulates at the mitotic centromere during prophase, persisting there until anaphase onset [3,4,5,6,7,8,9].

The centromere is the chromosomal locus upon which the kinetochore assembles, facilitating microtubule capture and chromosome segregation. While the DNA sequences found at centromeres vary widely between (and even within) species, epigenetic factors appear crucial in centromere identity. In most eukaryotes, centromeres are characterised by the presence of a histone H3 variant CENP-A. Nucleosomes containing CENP-A, interspersed with nucleosomes containing canonical H3, form a region of specialised centrochromatin upon which a network of centromere proteins assembles. This constitutive centromere-associated network (CCAN) is present throughout the cell cycle, but varies in its complexity across eukaryotes, from 16/17 different proteins in vertebrates, to a highly slimmed down version in *Drosophila* [10]. The highly interdependent proteins of the CCAN form a specialised domain that, during M phase, transiently recruits the outer kinetochore complex (the KMN network) that captures microtubules [11].

Both enzymatic and structural roles for topo 2 at the centromere have been proposed. In metazoans, while cohesin is largely removed from chromosome arms during prophase, it is retained at sister centromeres until anaphase onset [12]. Consequently, centromeric topo 2 is essential in anaphase to remove residual catenations, with directionality being provided by the separation of sister chromatids [13]. The bulk of these catenations is removed in early anaphase, but some persist as ultrafine threads that can be seen extending between separating sister chromatids, often only disappearing in mid, or even late, anaphase [14]. More recently, evidence of a non-catalytic role for topo 2, in recruiting other proteins to the inner centromere region during mitosis, has been reported for *Xenopus* and *Saccharomyces cerevisiae* [15,16,17,18,19].

Topo 2 activity can be assayed using a topo 2 poison, such as etoposide, that traps the enzyme mid-cycle, stabilising the normally transient phosphotyrosyl bonds linking each subunit of the topo 2 homodimer to the 5′ termini of the staggered DNA break [20]. As a result, these topo 2-mediated double strand breaks (DSBs) can be recovered and their position in the genome mapped through pulsed-field gel electrophoresis (PFGE) and Southern blotting. This approach has established that topo 2 does not bind and cleave all sites in the genome equally [21,22,23,24]. One preferred site of cleavage, identified in a few eukaryotes, is the centromere. This was first observed for the human Y centromere [25], and has since been extended to several other human α-satellite DNA-based centromeres [8,26], and to centromere regions in unicellular protozoa—specifically, the Kinetoplastids *Trypanosoma brucei* [27,28,29] and *Trypanosoma cruzi* [30], and the Apicomplexans *Plasmodium* [31,32] and *Toxoplasma gondii* [33].

To determine whether centromeric topo 2 cleavage activity extends beyond humans and unicellular protozoans, we have assayed the presence of this activity at centromeres of the fission yeast *Schizosaccharomcyes pombe* (*S. pombe*), the metazoan *Drosophila melanogaster* (*D. melanogaster*) and in another vertebrate species, *Gallus gallus* (chicken). The detection of centromeric topo 2 cleavage sites in these diverse eukaryotes underlines the fundamental and highly conserved nature of the active centromere “mark” recognised by topo 2.

## 2. Results

### 2.1. Topo 2 Cleavage within Centromeres of S. pombe

The fission yeast *S. pombe* has a monocentric chromosome architecture and is a powerful model system for the study of regional, CENP-A-based, centromeres. The three endogenous centromeres of *S. pombe* have similar overall DNA structures [34]. There is a central core (cnt) of 4–7 kb, surrounded by innermost repeats (imr) and outer repeats (otr). CEN 1 and CEN 3 share a 3.3 kb element (tm) in their central core (99% identical), while CEN 2 has a ~1.5 kb sequence in its central core that is similar to tm (48% identical). Each centromere has unique innermost repeats that are inverted relative to each other. The otr sequences show a high degree of sequence homology across the three centromeres, but differ in their number, orientation and organisation. tRNA genes (single, or as clusters) are present at all three centromeres. *S. pombe* centromeres have two distinct chromatin domains: the central core (where the CENP-A orthologue Cnp1 and the constitutive centromere-associated protein network is assembled), and the heterochromatic pericentromeric outer repeats.

It has been reported previously that fission yeast cells are insensitive to etoposide, but sensitive to a more potent topo 2 poison, TOP53 [35,36]. Like etoposide, TOP53 is derived from 4′-demethylepipodophyllotoxin [37,38]. Initially we examined the impact of treating log-phase cultures for 7 h (~3 cell cycles) with 50 µg/mL (85 µM) of TOP53, or with various TOP53 analogues (ICP 110, 129, 151, 166, 174, 193, 203). When fixed and stained with DAPI, most treated cells had a normal morphology; however, in ICP 166- and TOP53-treated cultures most cells had an elongated phenotype, consistent with a G2/M cell cycle arrest. We therefore proceeded to assay centromeric topo 2-cleavage activity using TOP53. Analysis of high molecular weight (HMW) DNA extracted after TOP53 exposure of log-phase cells did not reveal any evidence of cleavage within centromeres. However, cleavage was detected when log-phase cells were spheroplasted prior to TOP53 treatment. A range of TOP53 concentrations was tested (125–1000 µg/mL) for 90 min; since all yielded similar results experiments were routinely undertaken using 500 µg/mL (850 µM).

**Figure 1:**
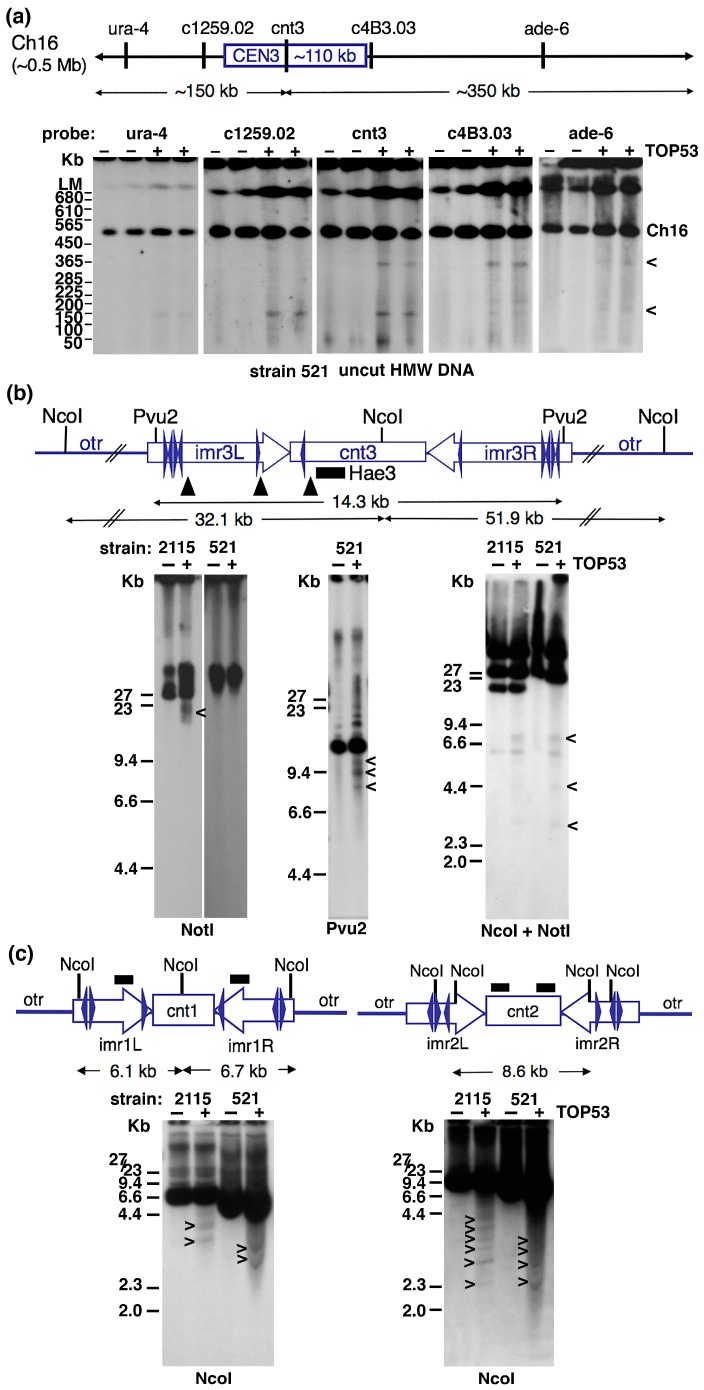
Molecular mapping of Topo 2 cleavage sites within centromeres of *S. pombe*. (**a**) Spheroplasted cells from strains #521 and #2115 were treated with solvent (H_2_O) or TOP53. Uncut HMW DNA from strain #521 was resolved by PFGE and Southern blotting performed. The 0.5 Mb linear minichromosome (Ch16) present in this strain carries the whole 110 kb CEN3 region, and can be resolved in the absence of restriction enzyme digestion. DNA probes from across the minichromosome revealed the presence of additional TOP53-specific signals. (**b**) Higher resolution mapping of topo 2-cleavage sites within the central core region of CEN3 using strains #521 and #2115. Like #521, strain #2115 carries a CEN3-based minichromosome in addition to the endogenous chromosomes. The minichromosome in strain #2115 is a 36 kb circular structure (CM3112) that retains the whole of the central core domain (cnt3, imr3L and imr3R) but this is flanked by only one set of outer repeats. CM3112 is linearised by digestion with NotI, which cuts at a single site within the minichromosome’s plasmid backbone. NotI does not cut within any of the endogenous centromeres. Pvu2 and NcoI sites within the central region of CEN3 are indicated. The DNA probe from the CEN3 cnt3 region (subclone Hae3) is specific for CEN3 (and does not cross react with CEN1). (**c**) Mapping of cleavage sites within the central regions of CEN1 and CEN2, using NcoI–cut DNA and centromere-specific DNA probes (originating from imr1, or cnt2, respectively). In (**b**,**c**), the position of tRNA genes within the central core and innermost repeat regions is indicated by blue arrowheads. TOP53-specific signals are indicated by “<”. Where possible, the approximate positions of the topo 2-mediated cleavage sites are indicated by black arrowheads on the schematic of the relevant centromere.

To facilitate analysis two strains, each carrying an additional CEN3-based minichromosome, were used: #521 and #2115 (Table 1). Such strains allow cleavage activity within the minichromosome-carrying centromere to be assayed directly on uncut high molecular weight (HMW) DNA. Figure 1a shows results obtained for strain #521, which has a ~0.5 Mb linear minichromosome (Ch16) that retains the whole of centromere 3 (~110 kb) [39,40]. Probe DNAs from across the minichromosome (Table 2) revealed several TOP53-specific signals. Notably probes from the left-hand side of the minichromosome (ura-4 and c1259.02) detected a novel band of ~150 kb, while a cnt3-specific DNA probe (Hae3) hybridised to two novel bands of ~150 and 350 kb. Probe DNAs from the right side of the minichromosome, c4B3.03, ade-6 also revealed TOP53-specific signals of ~150 and 350 kb. Probe c4B3.03 detected an additional band of ~200 kb. Overall these signals suggest that, in a significant proportion of cells, TOP53 traps DSBs generated by topo 2 within CEN3, cleaving the minichromosome into two fragments of ~150 and 350 kb. The data also suggest the presence of additional, non-centromeric, cleavage sites within the right-hand side of the minichromosome.

Topo 2 cleavage sites within CEN3 were examined by restriction enzyme mapping (Figure 1b). Consistent with the lack of sites for the restriction enzyme NotI within the 146 kb CEN3 contig available through PomBase (https://www.pombase.org), resolution of Not1-digested DNA by standard gel electrophoresis and Southern blot hybridisation with the Cnt3-specific DNA probe yields signals >30 kb (the limit of mobility). In strain #521 (carrying full sized copies of CEN3 on both endogenous chromosome 3 and on minichromosome Ch16), no TOP53-specific signals were detected in NotI-digested DNA. However, for strain #2115 a broad signal of ~20 kb is observed. In addition to the normal endogenous chromosomes, this strain carries a 36 kb circular minichromosome (CM3112) with its centromeric DNA limited to the central core of CEN3 and one set of outer repeats. CM3112 has a single NotI site within its plasmid backbone [41], accounting for the presence of a second signal >30 kb compared with strain #521. The TOP53-specific signal detected in this strain is consistent with DSBs trapped within the centrally-located CEN3 domain on the NotI-linearised 36 kb minichromosome (Figure 1b).

Higher resolution mapping of cleavage sites within the ~15 kb central core region (the cnt and imr domains) of CEN3 (with the signals originating from both the endogenous and minichromosome copies of this centromere), using PvuII and NcoI, revealed multiple novel TOP53 signals (Figure 1b). Cleavage activity could be mapped to three sites on the left-hand side of the central core domain. Whether cleavage is mirrored at the equivalent sites to the right side of the central core could not be ascertained, due to a lack of specific DNA probes. However, the fact that it is possible to detect both the 150 and 350 kb cleavage products of linear minichromosome Ch16 using the single CEN3-specific cnt3 subclone, Hae3 (Figure 1a) shows that it is possible to trap topo 2-mediated DSBs in both the left, and right, sides of the central core domain. 

The same mapping approach was then used to examine cleavage within CEN1 and CEN2 of the endogenous chromosomes in the 521 and 2115 strains. At both centromeres, multiple TOP53-specific signals were detected within the central core regions (Figure 1c).

Many studies have analysed chromatin structure at centromeres in *S. pombe* using MNase digestion and have reported an unusual chromatin organisation in the central core, that appears to lack regularly spaced nucleosomes [42,43]. In this study we have used a lower resolution in vivo approach that traps topo 2-mediated DSBs. While for CEN3 and CEN1 the parts of the central core regions to which cleavage sites have been mapped include tRNA genes, for CEN2 novel TOP53-induced fragments were detected within a region lacking tRNA genes, indicating that at least some of this centromeric topo 2-cleavage activity is independent of the presence of tRNA genes.

### 2.2. Topo 2 Cleavage within Drosophila Centromere 3

In *D. melanogaster*, each of the five endogenous centromeres contains different, and multiple, satellite DNAs, and the organisation of these repeated DNAs and their relationship (if any) to localisation of the histone H3 variant CENP-A (Cid in *Drosophila*), the essential epigenetic mark of the centromere in most eukaryotes, is unclear. Thus, while topo 2 cleavage has been reported within the 359 bp satellite III DNA that maps to the primary constriction of the *Drosophila* X chromosome [21], how this DNA relates to the core centromere has not been determined. In 2015 the first detailed physical map of an endogenous *Drosophila* centromere was reported [44]. The centromeric region of chromosome 3 (cytological region h53) contains two extensive and adjacent blocks of dodeca satellite repeat, which partially co-localise with CENP-A on polytene chromosomes and on extended chromatin fibres [44,45]. Cytological mapping and PFGE revealed that the dodeca repeat is juxtaposed with an extensive 10 bp satellite array, which extends from the primary constriction into chromosome arm 3 L (region h52), leading Garavis and colleagues to conclude that the centromere lies within the block I of the dodeca satellite DNA.

**Figure 2:**
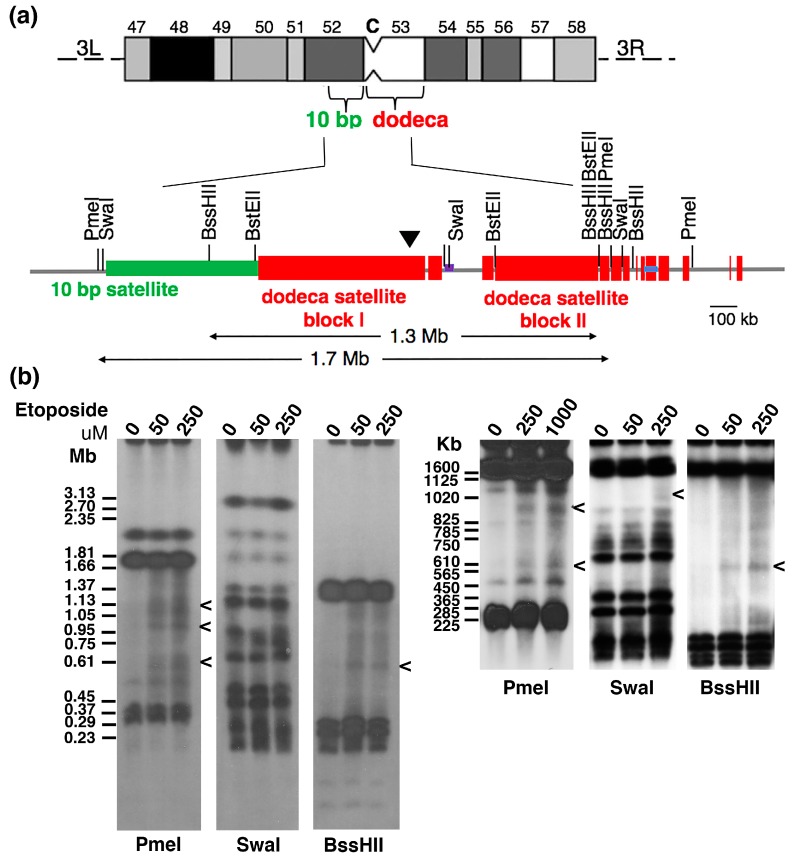
Molecular mapping of Topo 2 cleavage sites within centromere 3 of *Drosophila* Kc cells. (**a**) The physical map of the centromere 3 region is a modified version of the published physical map of centromere 3 based on the *D. melanogaster* red e strain [44], that takes into account PFGE and Southern blot hybridisation data obtained using the dodeca probe on DNA from Kc cells. (**b**) Asynchronously-growing Kc cells were treated with solvent (DMSO) or etoposide (at 50–1000 µM as indicated) for 60 min. The cells were then embedded in agarose and the HMW DNA digested with PmeI, SwaI or BssHII, resolved by PFGE (using various parameters) and Southern blot hybridisation undertaken using dodeca probe DNA. Etoposide-specific signals are indicated by “<“. The approximate position of topo 2-mediated DSBs is indicated by the black arrowhead on the physical map.

Using the published physical map based on the *D. melanogaster* isogenic red e strain [44,46], we examined the dodeca region in *Drosophila* Kc cells for evidence of topo 2 cleavage (Figure 2a). Asynchronously-growing Kc cells were exposed to etoposide (0, 50, 250 or 1000 µM) for 60 min before embedding them in agarose. PFGE, of SwaI, PmeI or BssHII-digested DNA and Southern blotting, using the dodeca DNA probe, placed DSBs from trapped cleavage complexes within the dodeca region. The complexity of the dodeca hybridisation pattern makes precise assignment of the cleavage sites within this domain challenging, but is consistent with cleavage within dodeca satellite block I (Figure 2b).

### 2.3. Topo 2 Cleavage within Satellite and Non-Satellite DNA-Based Centromeres in Chicken DT40 Cells

**Figure 3:**
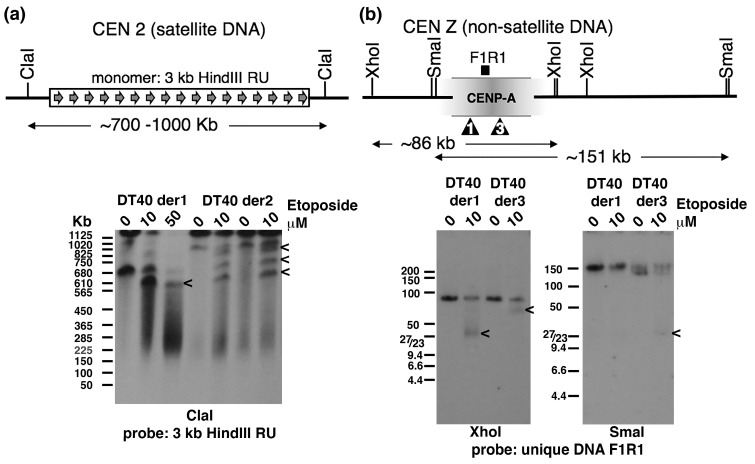
Mapping topo 2 cleavage sites within satellite and non-satellite DNA-based centromeres in chicken DT40 cells. (**a**) A schematic showing the ClaI sites flanking the satellite DNA array which forms the centromere of chromosome 2. The basic repeat unit, a 3 kb HindIII fragment, was used as a centromere 2-specific DNA probe on ClaI digested DNA from two DT40-based cell lines (der1 and der2, see the Methods for a full description) after treatment with etoposide, or solvent (DMSO). (**b**) A physical map of the region around the CENP-A-associated DNA of the Z centromere, based on the DT40 cell line characterised in [47]. Follow-up studies revealed that the size and precise position of the CENP-A domain varies across different wild type DT40 subclones, with the central position drifting within a 16.8 kb region, and the overall domain size averaging 33.4 ± 5.1 kb [48]. Probe DNA F1R1 is a 1.8 kb unique sequence from within the ~30 kb CENP-A-associated region identified by Shang et al. in 2010. The approximate locations of etoposide-trapped DSBs in CEN Z for the two DT40-derived lines are indicated by the arrowheads. Etoposide-specific signals are indicated by “<”.

It has been demonstrated that in chickens most macrochromosomes have centromeres with homogeneous, chromosome-specific, satellite repeat arrays that span hundreds of kilobases. In contrast, the centromeres of chromosomes 5, 27 and the Z lack satellite DNAs, and instead have non-satellite repeat DNA sequences spanning only ~30 kb [47]. To examine topo 2 cleavage activity within both types of chicken centromere, we focussed on the satellite DNA-based centromere of chromosome 2 and the non-satellite DNA-based centromere of the Z chromosome. Asynchronously-growing cells from various DT40-derived cell lines (detailed in the Methods section) were treated with etoposide (0, 10 or 50 µM), for 15 min, before being embedded in agarose. Following PFGE, ClaI-digested DNA was hybridised using the centromere 2 repeat unit (Figure 3a). Several etoposide-specific signals were detected: in DT40 der1, the starting ClaI band is ~700 kb with a strong etoposide-specific signal of ~600 kb also detected. In DT40 der2, the signal from starting array is at the gel’s limit of mobility (>1 Mb), with three novel bands (of ~ 900, 800 and 700 kb) generated by the topo 2 poison. Satellite DNA arrays are highly polymorphic and prone to changes in size and it appears that different DT40 subclones carry different sized arrays of this satellite DNA. In addition, the parental DT40 cell line is triploid for chromosome 2. Therefore, it is not possible to distinguish whether the various novel bands originate from different copies of chromosome 2 and/or from within the same array. Nevertheless, it is clear that etoposide robustly traps DSBs within this chicken centromeric satellite array, reminiscent of the situation reported for the α-satellite arrays at human centromeres. 

We then examined the much smaller, non-satellite repeat-based Z centromere, which is present as a single copy in DT40 cells. A physical map of the ~30 kb region of the chicken Z chromosome associated with CENP-A in DT40 [47,48] is shown in Figure 3b. It has been reported that the size of the CENP-A-associated domain, as well as its precise position on the Z chromosome, varies slightly between wild type DT40 subclones, indicating that the centromere can drift (across an ~15–20 kb region) during cell proliferation [48]. Although this centromere region lacks tandemly-repeated satellite DNA, it does contain other repeat DNAs, including extensive amounts of retrotransposon-derived sequences (which make up ~55% of CEN Z). A unique DNA sequence (F1R1) was identified and used to probe XhoI- and SmaI-cut DNA from two DT40-based cell lines briefly exposed to etoposide. For the XhoI-digested DNAs a novel, but different, band was detected in each of the two DT40 derivatives. In SmaI-digested DNA a novel signal was detected for DT40 der3; no etoposide-specific bands were apparent in DT40 der1. The likely position of the cleavage sites is indicated (Figure 3b). The mapping of cleavage sites to slightly different positions in these two DT40 derivatives may reflect centromere drift during cell proliferation. Nevertheless, the detection of etoposide-induced cleavage in this region is consistent with non-satellite DNA-based centromeres in chicken cells also displaying the “mark” recognised by topo 2.

### 2.4. CENP-I Depletion Reduces Centromeric Site-Specific Topo 2 Cleavage

We investigated whether disrupting the structure of the centromere impacts on topo 2 centromeric cleavage activity. DT40 is a powerful model system for assaying this phenotype because: (i) there are conditional null mutant DT40 lines available for many centromere proteins [49]; and (ii) centromeres in DT40 cells display robust topo 2-mediated DSBs upon brief exposure to low levels of etoposide [26]. We opted to quantitate cleavage activity within an extensively studied human α-satellite DNA-based centromere, present on a linear 2.7 Mb minichromosome. This “reporter” minichromosome was transferred into the DT40 mutant cell line M690 using microcell-mediated chromosome transfer. M690 expresses a doxycycline-regulatible CENP-I transgene as its only source of this essential, constitutive, core centromere protein [50]. The reporter minichromosome has a major topo 2 cleavage site within its α-satellite array, that can be resolved in otherwise undigested HMW DNA [8,26,51,52].

**Figure 4:**
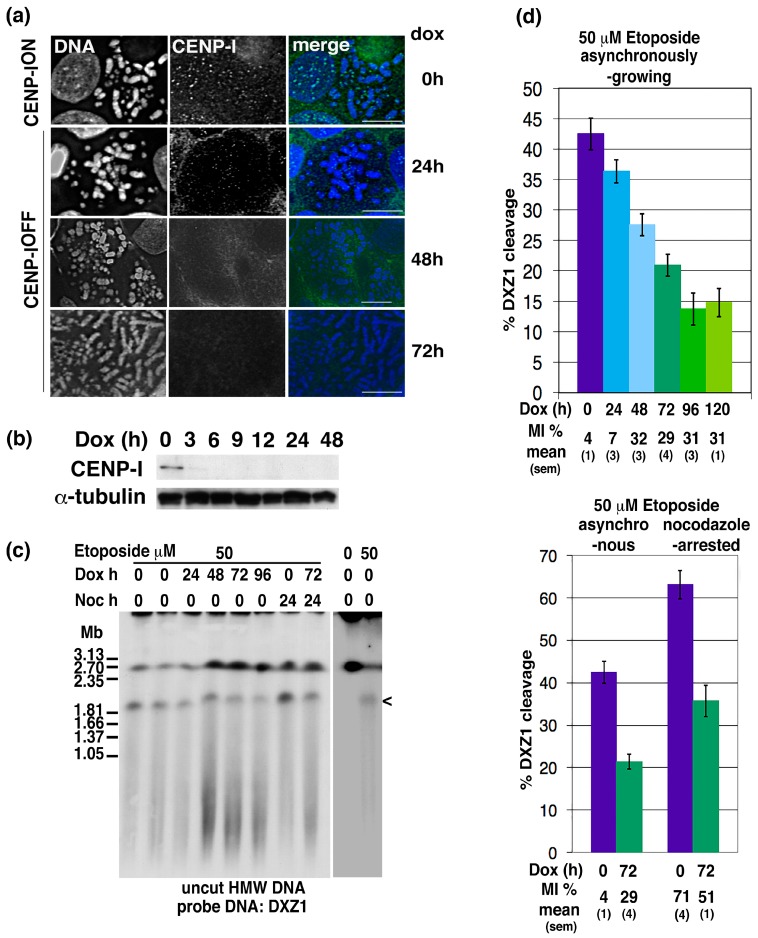
CENP-I influences levels of topo 2 cleavage activity at preferred sites within the centromere. (**a**) Indirect immunofluorescence for CENP-I in clone FA3M690-2, generated from a microcell-mediated chromosome transfer fusion between the DT40 CENP-I conditional knock out clone M6-90 and the human minichromosome:DT40 hybrid IKNFA3. The cell line retains the 2.7 Mb human X centromere (DXZ1)-based minichromosome (see (**c**)) in the CENP-I conditionally null mutant background. Cells were treated with doxycycline and samples prepared for IF over 72 h. Untreated cells, expressing CENP-I normally, are designated CENP-I^ON^, while those cells in which transcription of the CENP-I transgene is repressed, by doxycycline, are referred to as CENP-I^OFF^. DNA is stained with DAPI (blue in merged image). CENP-I was not detectable on chromosomes from cells treated with doxycycline for ≥24 h. Scale bar 10 µm. (**b**) Western blotting of whole cell extracts from clone FA3M690-3, with anti-CENP-I and anti-α-tubulin antibodies, sampled at various time points after the addition of doxycycline. (**c**) A representative Southern blot of a time course of the topo 2-PFGE cleavage assay. Cells were briefly exposed in vivo to etoposide (0 or 50 µM DMSO, 15 min) at various time points (0–96 h) after doxycycline addition and harvested into agarose blocks. Where indicated, cells were treated with nocodazole for 24 h prior to etoposide (or DMSO only) exposure. HMW DNA was resolved by PFGE, transferred and hybridised with the human DZX1 α-satellite DNA probe, specific for the minichromosome. The intact linear minichromosome is 2.7 Mb, with an etoposide-specific DXZ1 band of 1.85 Mb (indicated by the open “<”). (**d**) Summary of data from topo 2-PFGE cleavage assays. The amount of topo 2 cleavage within the centromeric region of the minichromosome was estimated by comparing the amount of signal for the 1.85 Mb cleavage fragment with the amount of the intact DXZ1 molecule in each sample. The effect of doxycycline exposure/CENP-I depletion on topo 2-DXZ1 cleavage levels was assayed for two hybrids multiple times, both on asynchronously-growing and nocodazole-arrested cell cultures. Presented are the mean values (s.e.m.) based on ≥6 independent experiments for each set of conditions. The mitotic index (MI) of each sample was assayed by indirect immunofluorescence for phosphorylated histone H3S10.

Depletion of CENP-I following doxycycline addition to two human X minichromosome:DT40 hybrid cell lines (FA3M690-2 and -3) was confirmed by indirect immunofluorescence and Western blotting (Figure 4a,b). Consistent with the findings of Nishihashi and colleagues, CENP-I turns over rapidly, becoming undetectable within 24 h [50]. Following etoposide exposure, HMW DNA was prepared and analysed by PFGE and Southern blotting using the α-satellite repeat unit (DXZ1). The human minichromosome migrates at ~2.7 Mb, with an etoposide-specific signal of ~1.85 Mb (a weaker etoposide signal of <1 Mb was often indistinguishable from the lower molecular weight smear) (Figure 4c). This is consistent with previous characterisation of etoposide-trapped DSBs within the centromere on this minichromosome [26,52]. The amount of topo 2 cleavage within the centromeric region of the minichromosome was estimated by comparing the signal for the 1.85 Mb cleavage fragment with that of the intact minichromosome in each sample. The effect of doxycycline exposure (CENP-I depletion) on topo 2-DXZ1 cleavage levels was assayed multiple times, both on asynchronously-growing and nocodazole-arrested cell cultures (Figure 4d). We found that the mitotic index of asynchronously-growing cells increased following doxycycline addition (Figure 4d), consistent with CENP-I depletion inducing a prometaphase arrest [50]. We have reported previously that, in wild type DT40 cells, the amount of centromeric topo 2-cleavage varies through the cell cycle, being highest in G2/M and lowest in G1/S [8]. However, despite CENP-I-depleted cells showing an increased MI, we observed a marked decrease in the amount of centromeric cleavage activity (as measured by levels of the 1.85 Mb DXZ1 fragment). This was observed both in etoposide-treated asynchronously-growing cultures and in cells exposed to nocodazole (*p* ≤ 0.005 when comparing the 0 and 72 h values within either set of samples) (Figure 4d).

## 3. Discussion

In this study, we show that site-specific cleavage by topo 2 within active centromeres is conserved widely across eukaryotes. As well as being detectable at satellite DNA-based centromeres in human, chicken and *Drosophila*, topo 2-mediated cleavage sites have also been identified in non-satellite repeat-based centromeres in *S. pombe* and chicken. This suggests that topo 2 must recognise a fundamental aspect of the centromere, such as the structure of the DNA or underlying chromatin.

We have shown previously that the level of topo 2 cleavage at specific sites within the centromere is cell cycle-dependent [8]. However, determining whether this reflects changes at the centromere locus during the cell cycle is not straightforward, due to the fact that topo 2 protein levels and activity are also cell cycle regulated (being higher in G2/M than in G1/S) [53]. In addition, in vertebrate cells the alpha isoform of topo 2 (which, unlike the beta isoform, is upregulated in proliferating cells) is recruited to mitotic chromatin and accumulates at centromeres [3,4,5,6,7,8,9]. In an earlier study, we reported that centromeric cleavage sites remain detectable when topo 2α levels are extensively depleted (<1% normal) [51]. While cleavage levels are reduced when topo 2α is depleted, it was only when both isoforms were depleted simultaneously that residual cleavage essentially disappeared [48]. Thus, although it is the alpha isoform that is recruited to mitotic chromatin and that accumulates at the centromere during M phase, both isoforms contribute to cleavage within the vertebrate centromere. This, together with the fact that in human cells topo 2 activity and cleavage are not detected at α-satellite arrays that are no longer associated with a centromere [6,25], allows topo 2 cleavage, the presence of a centromere, and topo 2 recruitment to be teased apart: site-specific cleavage requires an active centromere, but centromeric accumulation of topo 2 is not essential. While the robust levels of centromeric cleavage detected in asynchronously-growing vertebrate cells indicate that susceptibility of these sites to topo 2-mediated cleavage is not restricted to M-phase (and hence unlikely to be linked to assembly of the mature kinetochore complex) whether the centromere-associated mark being recognised by topo 2 is constitutive, or cell cycle-regulated, remains unclear.

Whatever aspect of the centromere locus is recognised by topo 2 it is determined by more than simply primary DNA sequence. As discussed earlier, in human cells topo 2 activity is associated with centromere-active, but not inactive, α-satellite arrays [6,25]. Moreover, it has been demonstrated that, while such satellite DNA arrays are often very extensive (hundreds, or even thousands, of kbps), topo 2 preferentially mediates cleavage within a highly restricted region of <50 kb which, based on immunofluorescence on stretched chromosomes and extended chromatin preparations, co-localises with the part of the array occupied by centromere proteins [25,26]. Similarly, for the non-satellite repeat DNA-based centromeres examined in this study (the chicken Z chromosome, and in *S. pombe*), where centromere proteins have been mapped at much higher resolution than is possible on homogeneous arrays of satellite DNA [34,47,48], topo 2 cleavage sites co-localise with the core centromeric chromatin.

DT40 cells have provided a powerful model system with which to assay centromeric topo 2-mediated DSBs because of the precise nature of the centromeric cleavage, which generates discrete signals reminiscent of a restriction enzyme. This has been observed irrespective of whether the activity was monitored at endogenous chicken centromeres, or at human α-satellite based centromeres transferred into the DT40 cell background. It may reflect the fact that centromeres in chicken are much more compact than those in human cells, as revealed both by the size of the CENP-A chromatin domain (spanning ~50 kb in chicken compared to ~500 kb or more in human cells) and in the number of microtubules captured by the kinetochore (estimated to be in the range of 3–7 microtubules per DT40 kinetochore, four- to five-fold fewer than for other metazoan kinetochores examined, including human) [47,54,55]. In an earlier study, we observed that, when mapping topo 2 cleavage sites within a series of human X centromere-derived minichromosomes generated in the DT40 cell background, in some clones, the position of cleavage had shifted slightly relative to that of the parental minichromosome [26]. Recently, Hori and colleagues reported that the extent and position of the Z centromere, as defined by the DNA associated with CENP-A, could shift slightly during cell proliferation [56]. Our observations on the human X centromere locus, as well as the finding, in the current study, that the position of topo 2 cleavage within the chicken Z centromere differed between two DT40-derived cell lines, argues that the region of topo 2 hypersensitivity tracks any drift in the position of the centromere relative to the underlying DNA.

To investigate further the role of constitutive centromere proteins in the susceptibility of this locus to topo 2 cleavage we focussed on CENP-I. CENP-I (Mis 6) is part of the CCAN in vertebrates and in *S. pombe* [50,57]. An essential protein, CENP-I forms a subcomplex with CENP-H, CENP-K and CENP-M, and its depletion disrupts the localisation of all members of the subcomplex [54]. Moreover, since the CENP-I/H/K/M subcomplex interacts with the other subcomplexes in the CCAN (CENP-C, CENP-L/N, CENP-T/W/S/X and CENP-O/P/Q/R/U), the presence of CENP-I is essential for maintenance of the of the inner centromere [49]. We show that depletion of CENP-I reduces levels of topo 2-mediated cleavage at sites within the vertebrate centromere. This contrasts to condensin depletion, where no impact on cleavage levels was observed [52]. This indicates that the aspect of the centromere that renders sites within it hypersensitive to topo 2 is unaffected by general disruption of mitotic chromatin structure (and loss of the normal axial distribution of topo 2), but is dependent on the integrity of the inner centromere.

What is it about the presence of a centromere that confers precise sites with hypersensitivity to topo 2 cleavage? Reports from others have identified centromeric topo 2 cleavage sites in various unicellular protozoa: trypanosomes, plasmodium and toxoplasma [27,28,29,30,31,32,33]. While candidate CENP-A orthologues have been identified in plasmodium and toxoplasma, CENP-A has not been identified in trypanosomes [58]. Indeed, it has recently been shown that, while trypanosomes have distantly-related homologues of some outer kinetochore proteins, a novel set of centromere proteins is present, that appears completely unrelated to the CENPs at conventional eukaryotic centromeres [59,60]. This argues for the centromere mark recognised by topo 2 not being linked specifically to CENP-A and the CCAN proteins of conventional eukaryotic centromeres, but to something more fundamental.

Although epigenetic factors, such as CENP-A, appear critical to centromere identity in many eukaryotes, the underlying DNA sequence also has a role to play. While the DNA sequences found at eukaryotic centromeres vary widely between species (and even within some species, like chicken), suggesting that they have not been selected for their primary DNA sequence, several studies have reported that diverse centromeric DNA sequences are enriched for dyad symmetries and for the propensity to form unusual DNA structures. In particular, the potential of centromeric DNAs to form intramolecular hairpin structures has been reported for primates, mouse, chicken, budding and fission yeasts and for the dodeca satellite-based *Drosophila* centromere [44,61,62,63]. If non-canonical secondary structures form specifically at active centromeres due to the torsional stress that arises within these domains, such non-B DNA structures may be the “mark” recognised by topo 2. In vitro studies have demonstrated that hairpins, including those formed from α-satellite DNA, can act as topo 2 substrates [64,65,66]. Such secondary structure might be induced by the binding of constitutive centromere proteins (such as CENP-B [63,67] or the CENP-T-W-S-X complex [68]), or could be triggered within the centromere in response to replication, or transcription, of this domain. RNA pol II transcription has been detected at human centromeres, both in interphase and in M-phase itself [69,70,71,72,73] and shown to be functionally important at fission yeast centromeres [74,75] and in *Drosophila* [76,77]. Alternatively, DNA entanglements and torsional stress arising during replication may be hard to resolve until cohesin is removed at anaphase onset [13,78].

While the accumulation of topo 2 at the centromere in M phase clearly has an essential function in resolving residual catenations during early anaphase, it is of interest to consider whether this site-specific cleavage has additional functional consequences for the core centromere domain itself, or whether it is simply a reflection of torsional stress. We have reported previously that depletion of topo 2α in a conditional null mutant human cell line had no obvious effect on the centromere/kinetochore over a 5 day period following transcriptional repression and gradual turnover of pre-existing protein [51]. However, the essential activities of topo 2 in shaping mitotic chromosomes, and in the separation of sister chromatids, may mask any centromere-specific role. Given that CENP-A (together with some other constitutive centromere proteins) has been reported to be recruited rapidly to DSBs in DNA [79], it is possible that topo 2 cleavage activity within the core centromere plays a role in priming the region for CENP-A recruitment. Alternatively, topo 2 cleavage might be required to relax DNA supercoils in non-coding RNA loci within centromeres during transcription [72,80]. On an evolutionary timescale, topo 2-mediated DSBs may promote centromeric-sister chromatid exchange (C-SCE) [81] and the evolution of centromeric satellite DNA arrays [82]. It seems likely that yet more roles for topo 2 remain to be identified.

## 4. Materials and Methods

### 4.1. S. pombe Strains

*S. pombe* media and growth protocols were essentially as described [83,84]. 

Sp521 carries a ~510 kb minichromosome (Ch16) that was generated from chromosome 3 and retains the whole of the ~143 kb CEN3 region [39,40]. Sp2115 carries a circular 36 kb CEN3-based minichromosome (CM3112) with the centromeric DNA limited to the central core of CEN3 and one set of outer repeats [41].

### 4.2. Insect and Vertebrate Cell Culture

The suspension insect cell line Kc167 (originating from female *D. melanogaster* embryos) was maintained according to the instructions provided by the Drosophila Genomics Resource Center, Indiana University. Standard DT40 culture conditions were used [85]. The endogenous chicken CEN 2 and CEN Z were examined in various DT40 derivatives: IKNFA3 (=DT40 der1), a somatic cell hybrid that is DT40 carrying a 2.7 Mb human X centromere-based minichromosomes [86]; ∆∆F5 (=DT40 der2), a DT40 conditional null CENP-A mutant [87]; and tTA5-5 (=DT40 der3), a DT40 conditional null CENP-H mutant [88]. The CENP-I conditional null mutant DT40 line, M690, carries a regulatable transgene as its only functional CENP-I locus [50]. Upon doxycycline (Sigma, St. Louis, MO, USA) addition (1 µg/mL) the CENP-I transgene is repressed, with protein levels are undetectable within 24 h. The reporter minichromosome was transferred into the recipient CENP-I DT40 mutant line using microcell-mediated cell fusion as described previously [85,89]. Microcells generated from the ~3 Mb human minichromome-DT40 line, IKNFA3 were fused with M690 and hybrid clones selected using 2 mg/mL G418SO_4_ (Geneticin, Invitrogen, Carlsbad, CA, USA), 2 mg/mL hygromycin (Calbiochem) (resistance cassettes present on the human minichromosome), 5 mM histidinol (Sigma), 0.5 µg/mL puromycin (Merck, Kenilworth, NJ, USA) and 1 mg/mL zeocin (Invitrogen) (resistance cassettes present in the null mutant line. Transfer of the human minichromosome was confirmed by PFGE of uncut HMW and Southern blot analysis with DZX1. Depletion of CENP-I in hybrid clones was confirmed by indirect immunofluorescence and by Western blotting. Once established, hybrids were routinely passaged in 300 µg/mL zeocin and 1 mg/mL hygromycin. For M-phase arrests, nocodazole (Sigma) was added to DT40 cells at a final concentration of 500 ng/mL.

### 4.3. Topoisomerase 2 Poisons

Etoposide (Sigma) was dissolved in 100% dimethylsulphoxide (DMSO) at 100 mM and stored in the dark at −20 °C. The inhibitor was added at a final concentration of 10–50 µM (as indicated) for 15 min at 37 °C (DT40 cells), or at 50–1000 µM for 60 min at 25 °C (for *Drosophila* Kc cells). TOP53 (HCl) was dissolved in H_2_O at 5 mg/mL and stored in the dark at −20 °C. Logarithmically-growing yeast cells were spheroplasted prior to treatment with TOP53. A range of concentrations was tested (50–1000 µg/mL, 85–1700 µM); since all yielded similar results on spheroplasts, most experiments were undertaken at 500 µg/mL (850 µM), for an incubation time of 90 min. In addition, various TOP53 derivatives were also tested for their effect on *S. pombe* in vivo. They were dissolved in DMSO, unless indicated otherwise: ICP110 (8b), ICP129 (8n), ICP151 (HCl) (solvent H_2_O) (13a), ICP166, ICP174 (8s), ICP193 (4a) and ICP203 (4g). In brackets are the original compound designations [37,38]. All have a similar molecular weight and were tested in vivo, alongside TOP53, at a final concentration of 50 µg/mL (85 µM). Why the topoisomerase 2 poison, its concentration and/or length of exposure, required to trap cleavage complexes efficiently varies across species and cell types is unclear, but may reflect differences in the import/efflux and/or metabolism of these drugs across species and cell types.

### 4.4. Molecular Biology

#### 4.4.1. Probe DNAs

DNA probes for *S. pombe* were obtained by PCR of strain 521 genomic DNA. Sequence information was obtained from PomBase (https://www.pombase.org/) and probe sequences designed to minimise cross-hybridisation and avoid tRNA genes. Centromere DNA probe sequence positions relate to the CEN contigs available in PomBase: CEN1: imr1—857 bp (CEN1 contig DNA sequence positions 34,627–35,500 and 41,542–42,398); CEN2: cnt2—a mix of two probes of 1057 and 1482 bps (CEN2 contig DNA sequence positions 50,175–51,232 and 53,628–55,110); and CEN3: cnt3(Hae3)—960 bp (DNA sequence position 66,083–67,043). 

Human X centromere: DXZ1, an X chromosome-specific α-satellite DNA 2 kb BamHI repeat unit from pSV_2_X5. *Gallus gallus* centromere 2: a CEN2-specific 3 kb HindIII satellite DNA repeat unit from p13-01. *Gallus gallus* Z centromere: most probe DNAs tested from within the CENP-A-associated DNA domain (AB556731.1 [44]) were found to work poorly on genomic DNA. However, one unique 1840 bp DNA sequence (designated F1R1) was identified that hybridises specifically, although weakly, to genomic DNA: this maps to the *Gallus gallus* genome sequence release 90.5 at position 42,757,179–42,759,018 and was generated using the following PCR primers: For: 5′-CCTAACCTGCTTCCACTGTTCG-3′ and Rev: 5′-ATCTGTGAGTCTTCTTTCAACGGG-3′. 

*Drosophila melanogaster* centromere 3: Dodeca satellite DNA was PCR-amplified from pBK6E218 [62].

#### 4.4.2. Gel Electrophoresis and Southern Blotting

DNA preparation, restriction enzyme digestion, gel electrophoresis, Southern blotting, hybridisation, and autoradiography were essentially as described [90]. To prepare *S. pombe* spheroplasts, logarithmically-growing cells were treated with zymolase (Zymo Research, Irvine, CA, USA) [84,91]. High molecular weight (HMW) DNA from yeast, insect and chicken cells was prepared in agarose blocks using standard methods and resolved by pulsed-field gel electrophoresis (PFGE) using either a Chef DRII (BioRad, ‎Hercules, CA, USA) or Biometra Rotaphor Type V (Analytik, Jena, Germany) apparatus [26]. DNA size markers used were: lambda DNA ladder, *S. cerevisiae* chromosomes, *H. wingei* chromosomes (BioRad). DNA was transferred to nylon membrane (Hybond N+, GE Healthcare Life Sciences, Little Chalfont, UK). Probe DNA was radioactively labelled by random-priming [92]. Southern blot hybridisations were carried out in a Hybaid oven and membranes washed at high stringency (0.2 × SSC/0.1% SDS at 65 °C).

### 4.5. Centromeric Topo 2-Cleavage Assay in DT40 Cells

Two independently generated human minichromosome, i.e. DT40 CENP-I conditional null hybrid clones (FA3M690-2 and -3), were used to assay topo 2-mediated cleavage activity within the centromere region. The amount of topo 2 cleavage within the centromeric region of the human X centromere (DXZ1)-based minichromosome was estimated by comparing the amount of signal for the 1.85 Mb cleavage fragment with the amount of the intact DXZ1 molecule in each sample (% cleavage = 1.85 DXZ1 signal/1.85 + 2.7 DXZ1 signal). Quantitation of the hybridisation signals was carried out on a Packard Instant Imager. Data are presented as the mean (s.e.m.). Statistical comparisons were performed using unpaired Student’s *t*-tests.

### 4.6. Indirect Immunofluorescence and Microscopy

Asynchronously-growing cells were treated for 15 min with Colcemid (KaryoMax, Invitrogen), resuspended in 75 mmol/L KCl and spun onto poly-l-lysine-coated slides (Cytospin, Shandon). Following fixation in absolute methanol, the cells were permeabilised in KCM (120 mmol/L KCl, 20 mmol/L NaCl, 10 mmol/L Tris.Cl, pH 8.0, 0.5 mol/L EDTA and 0.01% Triton X-100) and rabbit anti-chicken CENP-I antibody used at 1:1000 [50]. To estimate the mitotic index, cells were spun onto poly-l-lysine-coated slides in growth medium, fixed with 2% paraformaldehyde (ThermoFisher, Waltham, MA, USA) in PBS before permeabilisation in 0.1% Triton X-100 in PBS. The rabbit anti-histone H3S10ph antibody was used at 1:100 (Upstate). Secondary antibody, swine anti-rabbit FITC (Dako), was used at 1:100.

### 4.7. Western Blot Analysis

Total cell extracts were separated by SDS-polyacrylamide gel electrophoresis (PAGE) (XCell SureLock gel system, Invitrogen) and blotted onto PVDF-P membrane (Immobilon, Millipore, Billerica, MA, USA). After transfer, membranes were blocked with 5% skimmed milk in PBS and processed for ECL (ECL Plus, ThermoFisher) essentially as described [93]. Primary antibodies used were: rabbit anti-chicken CENP-I antibody (1:10,000 [50]) and mouse anti-α-tubulin (1:5000, ab7291 (DM1A), Abcam, Cambridge, UK). Secondary antibodies used were goat anti-rabbit HRP (1:4000) and goat anti-mouse HRP (1:4000).

## Figures and Tables

**Table 1:** Strain genotypes.

Strain	Genotype
#521	h− ade6-210 leu1-32 ura4-DS/E [Ch16 ade6-216 m23::ura4]
#2115	h+ ade6-704 his3-D1 leu1-32 ura4-D18 [CM3112 sup3e]

**Table 2:** PCR primers for *S. pombe* probe DNA.

*S. pombe* Locus	Forward Primer 5′-3′	Reverse Primer 5′-3′	Size (nt)
ura4	CAGAGGAAGCCTTTTTGCCAG	CCCATCTCACCGACCAACTTAG	618
c1259.02	CGTTCAATACGGAAACCTTACAG	GCAGAATCAGCAGAAGCAATGG	1041
c4B3.03	CGTTAGTGCTGAAAAGGATTCGG	AGGTGATTGCGACATTGACCC	1285
ade6	TCCTACTGCCATCAAAGCACTTG	GCAATAATCACACGCAACCCTC	846
imr1	TTTTTTACGCCACAATGTCGC	CAAACAGATACTTAGCCTCAAG	857
cnt2-1	TGCTATGTGGGTCTGTATTGCCTC	GGGGTTACCTTTTGCGATGG	1482
cnt2-2	TGAAGAAGCATGGTTAGTCCTGG	CCTGAACGGAACAAAACTCCTG	1058
cnt3	TCAGATTGTGGTCATAACAGTGC	TAGGTGAAGCGTAAGTGAGTGG	1642 ^1^

^1^ The 960 bp centromere 3-specific Hae3 probe fragment was isolated following digestion of the 1642 bp cnt3 PCR product with HaeIII.

## Abbreviations

Topo 2	Topoisomerase 2
CEN	Centromere
CCAN	Constitutive centromere-associated network
DSB	Double strand break
DMSO	Dimethylsulphoxide
PAGE	Polyacrylamide gel electrophoresis
PFGE	Pulsed-field gel electrophoresis
SDS	Sodium dodecyl sulphate
